# Population-level association of layperson cardiopulmonary resuscitation training with survival after out-of-hospital cardiac arrest: a systematic review and meta-analysis

**DOI:** 10.3389/fpubh.2026.1913160

**Published:** 2026-08-27

**Authors:** Pan Pan, Zhou YaXiong, Jiang YaoWen, Xia YiQin, Chen Yao

**Affiliations:** 1 Department of Emergency Medicine, Disaster Medical Center, West China Hospital, West China School of Medicine, Sichuan University, Chengdu, Sichuan, China; 2 Department of Emergency Medicine, West China Tianfu Hospital, Sichuan University, Chengdu, Sichuan, China

**Keywords:** bystander CPR, cardiopulmonary resuscitation training, meta-analysis, out-of-hospital cardiac arrest, survival outcome

## Abstract

**Background:**

Out-of-hospital cardiac arrest (OHCA) is a significant worldwide cause of death and is highly dependent upon bystander intervention. Layperson cardiopulmonary resuscitation (CPR) training has been widely promoted, but its population-level association with survival after out-of-hospital cardiac arrest remains incompletely characterized.

**Methods:**

This systematic review and meta-analysis was conducted according to PRISMA guidelines and registered in PROSPERO. PubMed, Embase, Web of science and Cochrane Library and major Chinese database were searched from inception to June 1, 2026. Studies evaluating the association between layperson CPR training and OHCA survival outcomes were included. The main outcome measure was survival to hospital discharge. Random-effects models were used for calculation of pooled odds ratios (ORs) and 95% confidence intervals (CIs). I^2^ statistics and Egger’s / Begg’s tests were used to evaluate heterogeneity and publication bias.

**Results:**

Nineteen observational studies comprising 159,378 participants from eight countries were included in the systematic review. Seventeen studies comprising 144,775 participants contributed to the training-modality-specific meta-analyses. Layperson CPR training was associated with higher survival to hospital discharge. In the training-modality-specific analyses, favorable associations were observed for both CPR-only training (OR = 1.74, 95% CI: 1.39–2.19) and CPR combined with AED training (OR = 1.42, 95% CI: 1.15–1.77). There was evidence of significant heterogeneity among the studies, but no evidence of publication bias.

**Conclusion:**

Layperson CPR training was associated with higher population-level survival after OHCA and should be viewed as one component of an integrated community resuscitation system.

**Systematic review registration:**

https://www.crd.york.ac.uk/prospero/record_view.phprecid=CRD420261479204.

## 1 Introduction

Out-of-hospital cardiac arrest (OHCA) is one of the most time-sensitive medical emergencies, and although emergency medical services (EMS) has evolved to improve survival rates, OHCA continues to have a low survival rate globally (1). There is currently evidence that most areas have a survival to hospital discharge of between 5% and 10% and that even lower figures may be found in low and middle-income areas (2). OHCA is time sensitive with irreversible neurological injury occurring in 4–6 min of circulatory arrest (3). Accordingly, the ‘chain of survival’ emphasizes rapid recognition of cardiac arrest and early initiation of CPR as key factors associated with survival.

Of all the steps in the chain of survival, bystander-initiated CPR has been and continues to be recognized as the most critical modifiable factor that is associated with survival. Previous epidemiological studies have shown that early CPR before EMS arrival is associated with higher rates of ROSC and survival to hospital discharge (4). Still, even with a high level of clinical consensus, implementation of bystander CPR (B-CPR) is suboptimal and very heterogeneous globally. Bystander CPR rates reported across systems range from < 20% to > 60%, with significant differences in public preparedness, education coverage, and integration into systems, depending on the maturity of the resuscitation system in the country (5, 6).

To address these gaps, many countries have implemented large-scale pre-hospital CPR training programmes for laypersons, including mandatory school-based instruction, workplace training, community mass-training campaigns, dispatcher-assisted CPR instruction, and simplified compression-only CPR delivered through conventional, digital, or mobile-health platforms (7). CPR training is also sometimes integrated with the education of automated external defibrillators (AEDs), to enhance the potential for early defibrillation. These interventions have been associated with increased delivery of bystander CPR, although the magnitude of their association with survival outcomes has varied across studies. The observed survival patterns may be related to training coverage, EMS response intervals, baseline cardiac arrest incidence, socioeconomic conditions, AED availability, and the organization of healthcare systems.

Although there is an increasing number of observational and quasi-experimental studies comparing pre-hospital CPR training interventions, evidence is still limited, and the methodological heterogeneity of the studies hinders the interpretation of the results. Previous studies have a wide range of designs, population, intensity of intervention and definitions of outcome, so there are varied conclusions about the size of the effect and even about whether it is positive or negative. Further, there are different levels of maturity in health care systems, EMS response systems, and bystander CPR training prevalence across studies that complicates direct comparisons. However, the population-level association between CPR training and survival remains incompletely characterized, particularly across settings with different EMS structures, baseline levels of community preparedness, and access to early defibrillation. There is thus a need for a more complete synthesis of the available evidence to bring together the fragmented evidence and for a better understanding of the consistency and strength of associations reported in various settings.

Thus, a systematic review and meta-analysis was performed to give a holistic assessment of the existing evidence for the relationship between community-based CPR training and post OHCA survival outcomes. Particular attention was given to variation in the observed associations according to training modality, geographic setting, and publication period. This systematic review and evidence synthesis provides an overview of the available evidence and examines important sources of heterogeneity that may contribute to variation in the observed associations. The findings may inform public health policy and the population-level design, implementation, and scaling of CPR training programmes.

## 2 Materials and methods

### 2.1 Study design and reporting standards

This systematic review and meta-analysis was performed following the Preferred Reporting Items for Systematic Reviews and Meta-Analyses (PRISMA 2020) guidelines to ensure methodological rigor, transparency and methodological reproducibility. Study protocol has been registered in PROSPERO international database before data extraction (registration number: XXXX). Methodological procedures were carried out according to Cochrane Handbook for Systematic Reviews of Interventions guideline, standardizing procedures of study selection, data extraction, risk of bias assessment, and quantitative synthesis. Because CPR training programmes are commonly implemented at the population level and relatively few randomized studies were available, both experimental and observational study designs were eligible to capture the available real-world evidence.

### 2.2 Research question and PICOS framework

PICOs eligibility and analytical framework were used to structure the research question. These were community-based people with OHCA, a condition that is associated with high mortality and time-sensitive interventions. The intervention was defined as any pre-hospital and/or community based training in CPR whether instructor-led, school based, workplace based, dispatcher-assisted training, multimedia training, online training, hands-only CPR, or CPR/AED training that was offered to laypersons. The comparator was people in populations that did not have structured CPR training exposure or baseline conditions before the CPR training programs were implemented. Primary outcome was survival to hospital discharge as this represented clinically meaningful long term resuscitation success and secondarily return of spontaneous circulation (ROSC) and neurologically favourable survival if reported. Due to the pragmatic and population level nature of CPR training interventions, study designs considered eligible included randomized controlled trials, cluster randomized trials, quasi-experimental studies, cohort studies, and interrupted time-series analyses.

### 2.3 Literature search strategy

A systematic search of the literature was performed in PubMed, Embase, Web of Science Core Collection, Cochrane Library, China National Knowledge Infrastructure (CNKI), Wanfang Data, VIP Database, and the Chinese Biomedical Literature Database (CBM) from the inception of the databases until June 1, 2026. We used a combination of the controlled vocabulary (MeSH/Emtree) and free text terms for cardiac arrest and CPR training, such as “out-of-hospital cardiac arrest,” “cardiopulmonary resuscitation,” “CPR training,” “bystander CPR,” “community CPR education,” “public training,” and “survival outcome.” Boolean operators (AND/OR) were used to maximize sensitivity and specificity. All items retrieved were not restricted to any language and only English and Chinese language studies were analyzed. To limit publication bias, reference lists of all the eligible studies and relevant reviews were hand-searched to identify other studies.

### 2.4 Eligibility criteria

Using the PICOS framework, eligibility criteria were pre-defined. Studies were eligible if they evaluated layperson CPR training in pre-hospital or community settings by comparing outcomes between populations with and without training exposure or between periods before and after implementation of a training programme. Eligible studies needed to report at least one survival outcome after OHCA and had to be either a randomized controlled trial, cluster randomized trial, quasi-experimental design, cohort study, or time-series analysis published in a peer-reviewed journal. CPR training interventions included all forms of structured training, such as traditional instructor-led training, hands-only CPR training, dispatcher-assisted CPR training, training using multimedia, school- or workplace-based training, and CPR training that also included training in the use of an AED. Studies were excluded if they did not provide enough data to estimate an effect size (e.g., lack of total numbers of participants, or failure to report outcome data for the combined analysis); if they included duplicated or overlapping populations; or were case reports, narrative reviews, editorials, conference abstracts without full text, animal studies or simulation-only studies. Studies that only evaluated in-hospital CPR training for healthcare workers were not included. To ensure reliability and the reproducibility of the data, unpublished data, personal communications and manuscripts under review were not included, as per PRISMA and Cochrane methodological guidelines.

### 2.5 Study selection and data extraction

All retrieved records were put into NoteExpress software to be systematically managed and deduplicated. Study screening was performed by two independent reviewers in two phases: title/abstract screening, and full-text screening based on the eligibility criteria. Any disagreement was settled by discussion and where required a final senior adjudicator to ensure consensus. Study characteristics, population size, intervention type, comparator characteristics, outcome definitions, follow-up time points, reported effect measures, adjustment variables, and the numbers of outcome events and participants in each comparison group were independently extracted using a standardized data extraction form. Multivariable-adjusted ORs were prioritized when available. When a study reported more than one adjusted model, the estimate with the most comprehensive adjustment for clinically relevant baseline confounders was selected. When no adjusted OR was reported, an unadjusted OR was calculated from the available binary outcome data. Only one effect estimate from each study was included in a given pooled analysis to avoid double counting. Survival to hospital discharge was prioritized as the primary outcome; when it was unavailable, the closest prespecified longer-term survival outcome, such as 30-day survival, was selected. ROSC and neurologically favorable survival were considered secondary outcomes and were not combined with survival to hospital discharge in the same pooled estimate.

### 2.6 Quality assessment and risk of bias

The methodological quality and risk of bias of the included studies were assessed using the Newcastle–Ottawa Scale (NOS). The NOS was selected because the evidence base consisted predominantly of cohort and registry-based observational studies and because it provides a consistent assessment of three relevant dimensions: selection of study populations, comparability of exposure groups, and ascertainment of outcomes. The scale ranges from 0 to 9 points. For descriptive purposes, studies scoring ≥ 7 were categorized as high quality, those scoring 5–6 as moderate quality, and those scoring ≤ 4 as low quality. These categories were used to summarize the methodological characteristics of the included studies. ROBINS-I was also considered; however, because the included evidence comprised a mixture of population exposures, registry-based comparisons, and system-level programmes rather than uniformly defined non-randomized interventions, the NOS was selected to provide a consistent assessment across studies. Two reviewers independently evaluated each NOS item, and disagreements were resolved through discussion or consultation with a third reviewer.

### 2.7 Statistical analysis

Data were analyzed using Stata version 18.0. The primary summary measure was the OR with its 95% CI. Adjusted ORs were preferentially used when available; otherwise, unadjusted ORs and their standard errors were calculated from reported 2 × 2 event data. When studies reported an RR or HR rather than an OR, the reported measure was not treated as directly equivalent to an OR. An OR was calculated from the binary event data at the prespecified outcome time point when sufficient information was available. Study-specific ORs and confidence limits were transformed to the logarithmic scale, and the corresponding standard errors were derived from the 95% CIs. Log ORs were pooled using inverse-variance weighting. Cochran’s Q test and I^2^ statistic were used to examine between-study heterogeneity, and an I^2^ > 50% or *p* < 0.10 suggested that there was significant heterogeneity. Because clinical and methodological variation was anticipated across training programmes, populations, healthcare systems, and study designs, a random-effects model using the DerSimonian–Laird method was used for the primary analysis. Fixed-effect models were used for sensitivity analyses. Potential sources of heterogeneity were examined through subgroup analyses according to geographic region, training modality (CPR-only vs. CPR plus AED training), and publication period (≤2016 vs. >2016). The robustness of the pooled estimates was further assessed using leave-one-out sensitivity analyses, in which each study was sequentially excluded and the summary estimate was recalculated.

### 2.8 Publication bias assessment

Both the Egger’s regression test and Begg’s rank correlation test were used to assess for publication bias, using *p* < 0.05 as the level of significance. Funnel plots were manually examined for asymmetry where there were 10 or more studies. The complementary approaches were used to evaluate potential small-study effects and the robustness of pooled estimates, especially because most of the literature included were observational study designs.

### 2.9 Ethical considerations

No primary data was collected from animals or human beings, and this study was based on data already published. So no ethics approval or informed consent was necessary.

## 3 Results

### 3.1 Study selection

**Figure 1:**
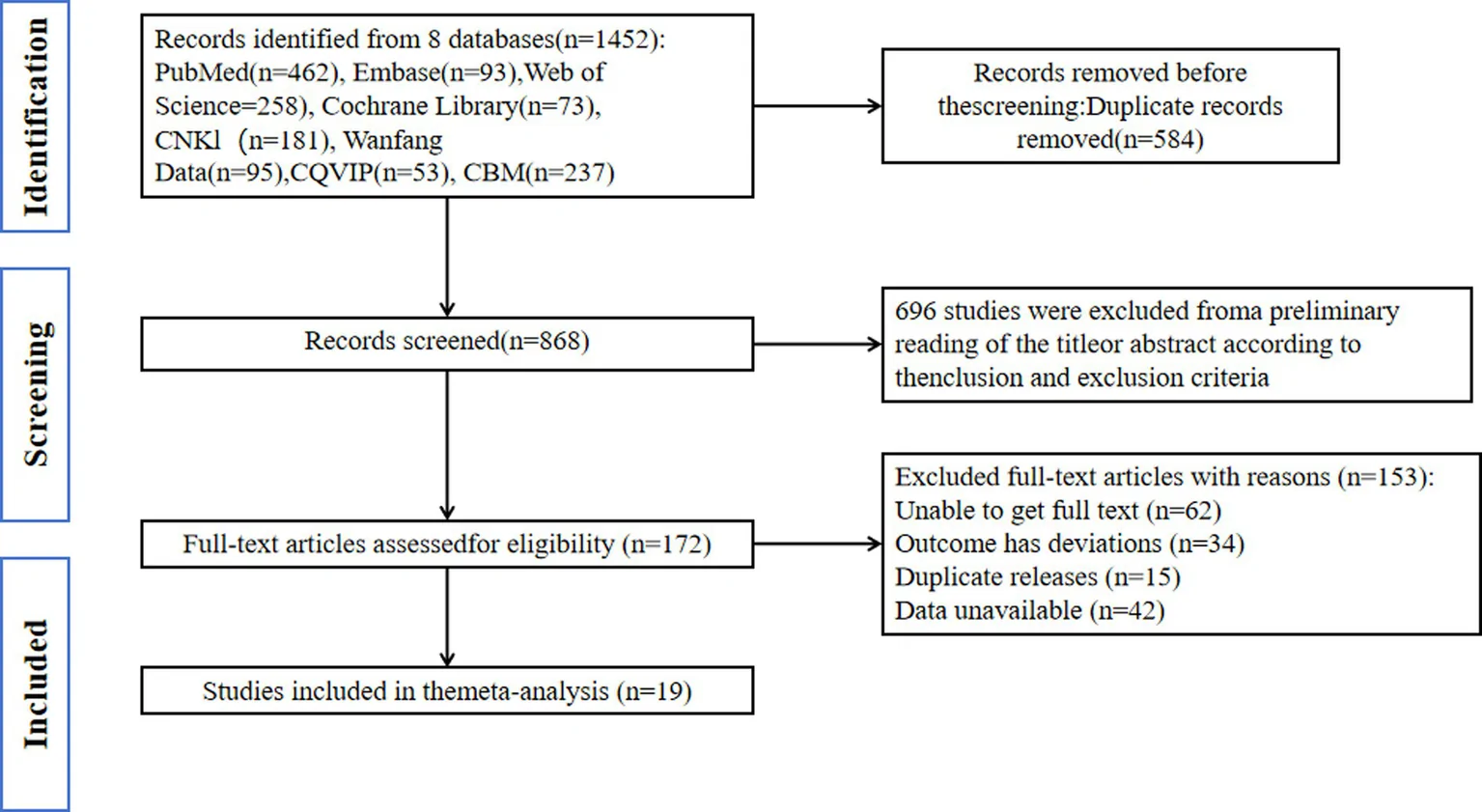
PRISMA 2020 flow diagram of study selection for the systematic review and meta-analysis of pre-hospital CPR training and OHCA survival outcomes.

Following assessment of 172 full-text articles, 19 studies (8–26) met the PICOS inclusion criteria and were included in the systematic review. Of these, 17 studies provided compatible data for the training-modality-specific quantitative synthesis. The remaining two studies were retained in the qualitative synthesis but were not included in the training-modality-specific meta-analysis because Li et al. (24) evaluated a bundled legislative and system-level programme incorporating public CPR training, AED promotion, and legal guidance that could not be assigned to a single training modality, whereas Fabianek et al. (25) primarily reported ROSC and hospital-admission outcomes and did not provide a compatible survival-to-discharge or equivalent longer-term survival estimate for quantitative pooling. The study selection process is shown in the flow diagram (Figure 1) and was done in accordance with PRISMA 2020 guidelines. There is a significant drop-off at the screening stage, largely because of the absence of reporting of survival outcomes, the absence of exposure to CPR training in the studies, or because studies were not population based.

### 3.2 Study characteristics and methodological quality

Table 1 shows the characteristics of the studies included in this evaluation. The studies ranged in publication date from 2005 to 2024, and took place in eight different countries, including across Europe, North America and Asia. This geographically wide spread encompasses significant heterogeneity with regard to emergency medical system (EMS) systems, CPR education and bystander intervention capacity, which may lead to heterogeneity in the estimates of effect.

**Table 1:** Characteristics of the included studies evaluating layperson CPR training and survival outcomes after OHCA.

First author	Year	Country/Region	Intervention/control, *n*	Training programme	NOS score
Herlitz et al. (2005) (8)	2005	Sweden	8319/21391	CPR	7
Lick et al. (2011) (9)	2011	USA	48/247	CPR + AED	7
Wissenberg et al. (2013) (10)	2013	Denmark	8741/4107	CPR	6
Malta Hansen et al. (2015) (11)	2015	Denmark	1145/699	CPR + AED	7
Lai et al. (2015) (12)	2015	Singapore	3025/2428	CPR	8
Ringh et al. (2015) (13)	2015	Sweden	306/361	CPR	7
Yu et al. (2016) (14)	2016	China	193/914	CPR + AED	7
Li et al. (2017) (15)	2017	China	11/106	CPR	6
Ge et al. (2017) (16)	2017	China	118/82	CPR	6
Ro et al. (2017) (17)	2017	South Korea	11,794/20706	CPR + AED	7
Uber et al. (2018) (18)	2018	USA	587/899	CPR	6
Li et al. (2019) (19)	2019	China	143/126	CPR	6
Blewer et al. (2020) (20)	2020	Singapore	3248/3529	CPR	7
Haskins et al. (2020) (21)	2020	Australia	3703/761	CPR + AED	7
Tay et al. (2020) (22)	2020	Singapore	361/880	CPR + AED	7
Zheng et al. (2023) (23)	2023	China	7121/35090	CPR	6
Li et al. (2024) (24)†	2024	China	5893/7858	System-level CPR + AED legislation	7
Fabianek et al. (2024) (25)‡	2024	Germany	101/751	Smartphone-dispatched CPR-trained volunteers	8
Zhang et al. (2024) (26)	2024	China	936/2650	CPR + AED	6

AED, automated external defibrillator; CPR, cardiopulmonary resuscitation; NOS, Newcastle–Ottawa Scale; OHCA, out-of-hospital cardiac arrest. ^†^Included in the qualitative synthesis only because the exposure was a bundled legislative and system-level programme incorporating public CPR training, AED promotion, and legal guidance and could not be assigned to a single training modality. ^‡^Included in the qualitative synthesis only because the study primarily reported ROSC and hospital-admission outcomes and did not provide a compatible discharge or longer-term survival estimate for the prespecified quantitative synthesis.

All of the studies included in the review were observational (prospective cohort studies, retrospective registry studies, and quasi-experimental population-based interventions). No randomized controlled trials were identified.

Intervention strategies were broken down into two groups: CPR-only intervention training and CPR and AED intervention training. A high degree of variability was noted between studies in terms of training intensity, delivery mode and target population, such as mandatory CPR training in schools, workplace training programs, community mass training campaigns, dispatcher-assisted CPR, and lay responder activation platforms via smart phones.

**Figure 2:**
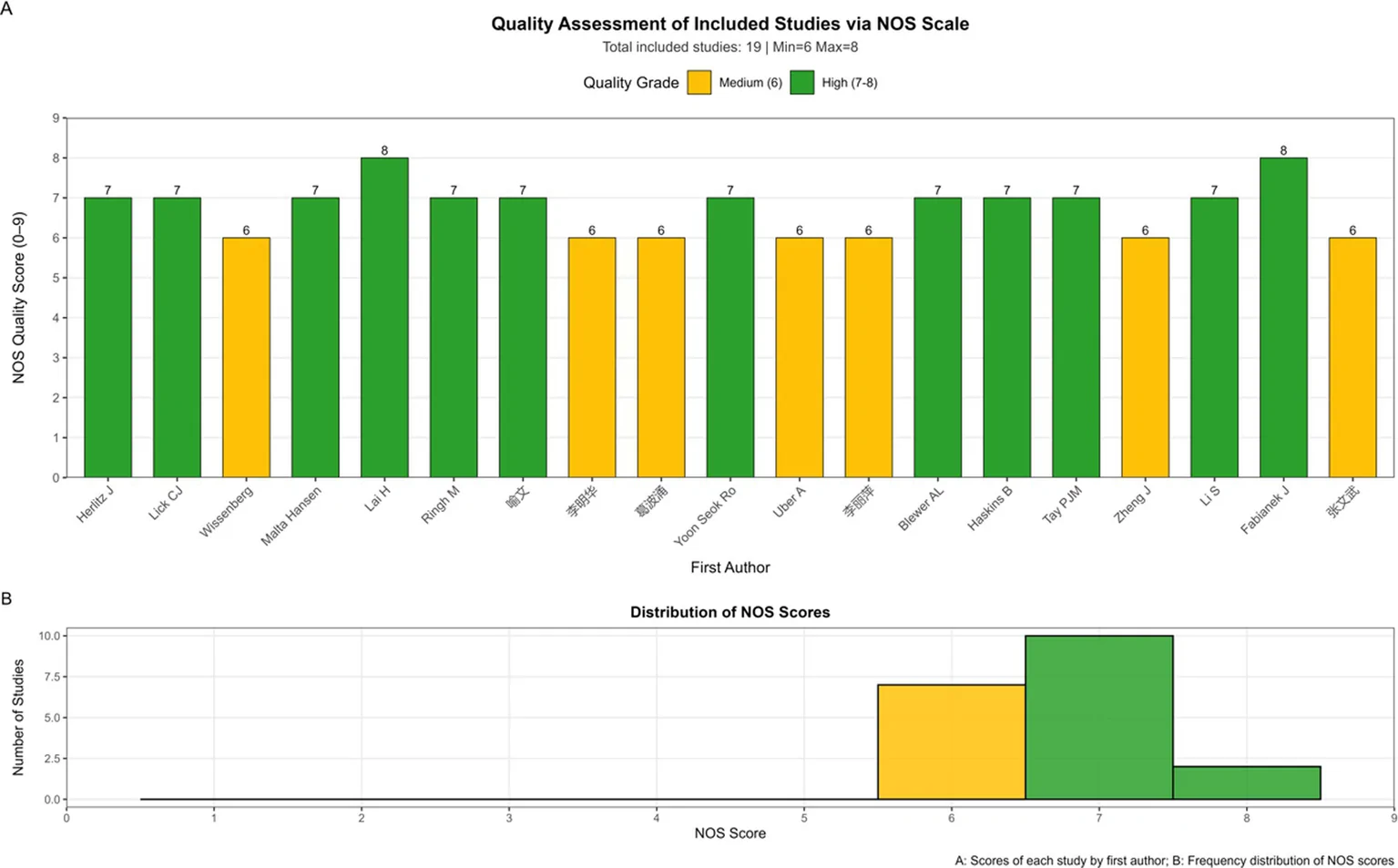
Newcastle–Ottawa Scale assessment of the included studies. **(A)** Individual NOS scores of the included studies, with studies categorized as moderate (5–6 points) or high (≥7 points) methodological quality. **(B)** Distribution of NOS scores among the included studies.

According to the predefined descriptive NOS categories, 12 studies scored ≥ 7 and seven scored 5–6. Outcome ascertainment was generally based on registry- or hospital-verified survival data. The NOS assessments of the included studies are presented in Table 2 and Figure 2.

**Table 2:** Methodological quality assessment of the included studies using the Newcastle–Ottawa Scale.

Study	NOS score	Descriptive category
Herlitz et al. (2005) (8)	7	High
Lick et al. (2011) (9)	7	High
Wissenberg et al. (2013) (10)	6	Moderate
Malta Hansen et al. (2015) (11)	7	High
Lai et al. (2015) (12)	8	High
Ringh et al. (2015) (13)	7	High
Yu et al. (2016) (14)	7	High
Li et al. (2017) (15)	6	Moderate
Ge et al. (2017) (16)	6	Moderate
Ro et al. (2017) (17)	7	High
Uber et al. (2018) (18)	6	Moderate
Li et al. (2019) (19)	6	Moderate
Blewer et al. (2020) (20)	7	High
Haskins et al. (2020) (21)	7	High
Tay et al. (2020) (22)	7	High
Zheng et al. (2023) (23)	6	Moderate
Li et al. (2024) (24)	7	High
Fabianek et al. (2024) (25)	8	High
Zhang et al. (2024) (26)	6	Moderate

NOS, Newcastle–Ottawa Scale. For descriptive purposes, scores ≥ 7 were categorized as high quality, scores of 5–6 as moderate quality, and scores ≤ 4 as low quality. These categories were not considered definitive domain-level risk-of-bias judgments.

Survival to hospital discharge was the preferred primary outcome. When this outcome was unavailable, 30-day survival or the closest prespecified longer-term survival measure was used. The survival outcomes were therefore clinically related but not completely uniform across all studies.

### 3.3 Pooled associations between layperson CPR training and survival outcomes

#### 3.3.1 CPR-only training and survival outcomes

**Figure 3:**
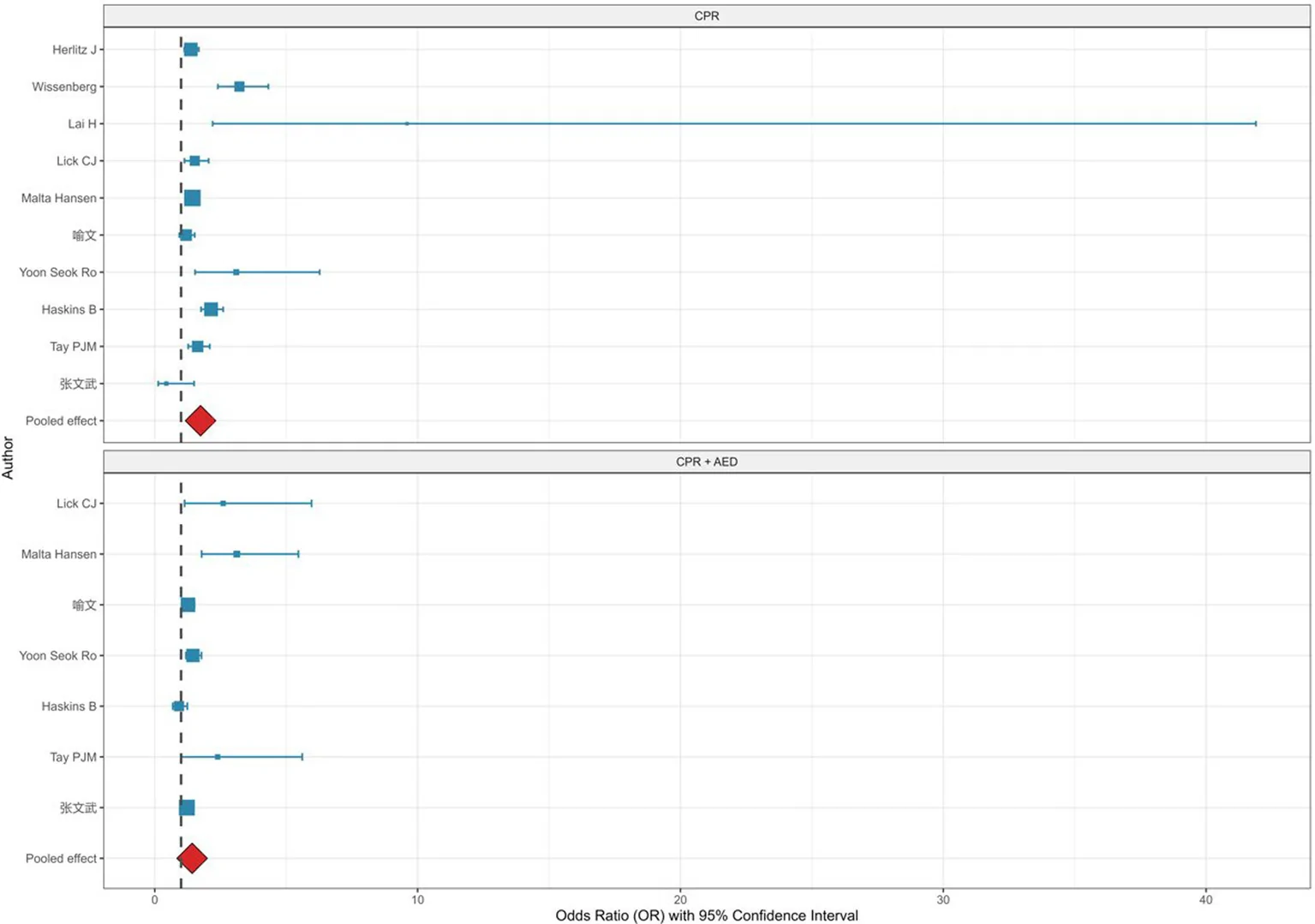
Forest plots of the association between layperson CPR training and survival outcomes after OHCA.

The CPR-only meta-analysis included 10 studies comprising 99,738 participants (Table 3). There was substantial heterogeneity (Q = 53.482, I^2^ = 83.2%, *p* < 0.001), suggesting that there was variation in effect sizes across populations and contexts of intervention. Thus, a random-effects model was used. Despite this heterogeneity, CPR-only training was significantly associated with higher survival to hospital discharge after OHCA (OR = 1.74, 95% CI: 1.39–2.19; Z = 4.78; *p* < 0.001), as illustrated in the forest plot (Figure 3). This association may reflect several interrelated pathways, including a greater likelihood of bystander intervention, a shorter no-flow interval, and earlier maintenance of circulation before EMS arrival. The magnitude of the pooled estimate indicates a clinically relevant population-level association between CPR-only training and survival.

**Table 3:** Pooled associations between layperson CPR training and survival outcomes after OHCA by training modality.

Training modality	No. of studies	No. of participants	Q	I^2^ (%)	*P* for heterogeneity	Pooled OR (95% CI)	*Z*	*p*
CPR-only training	10	99,738	53.482	83.2	< 0.001	1.74 (1.39–2.19)	4.78	<0.001
CPR plus AED training	7	45,037	21.053	71.5	0.0018	1.42 (1.15–1.77)	3.23	0.0012

OR, odds ratio; CI, confidence interval; CPR, cardiopulmonary resuscitation; AED, automated external defibrillator. Participant totals represent the combined intervention and control populations of the studies contributing to each analysis. Estimates were obtained using random-effects models. Seventeen studies comprising 144,775 participants contributed to the training-modality-specific quantitative synthesis.

#### 3.3.2 CPR combined with AED training and survival outcomes

A total of seven studies comprising 45,037 participants evaluated the association between combined CPR and AED training and survival outcomes (Table 3). There was also substantial heterogeneity (Q = 21.053, I^2^ = 71.5%, *p* = 0.0018) and a random-effects model was adopted. The pooled analysis showed that CPR plus AED training was significantly associated with higher survival to hospital discharge (OR = 1.42, 95% CI: 1.15–1.77; Z = 3.23; *p* = 0.0012) (Figure 3). The pooled estimate was numerically smaller than that observed for CPR-only training; however, this difference should not be interpreted as evidence that one training modality is superior to the other. Instead, it is probably a result of the difference in the accessibility of AEDs, the number of AEDs per person and real-world actual AED use rates that are still greatly dependent on regional infrastructure and public access defibrillation systems.

### 3.4 Subgroup analyses of the association between CPR training and survival outcomes

#### 3.4.1 Geographic variation and health system disparities

**Figure 4:**
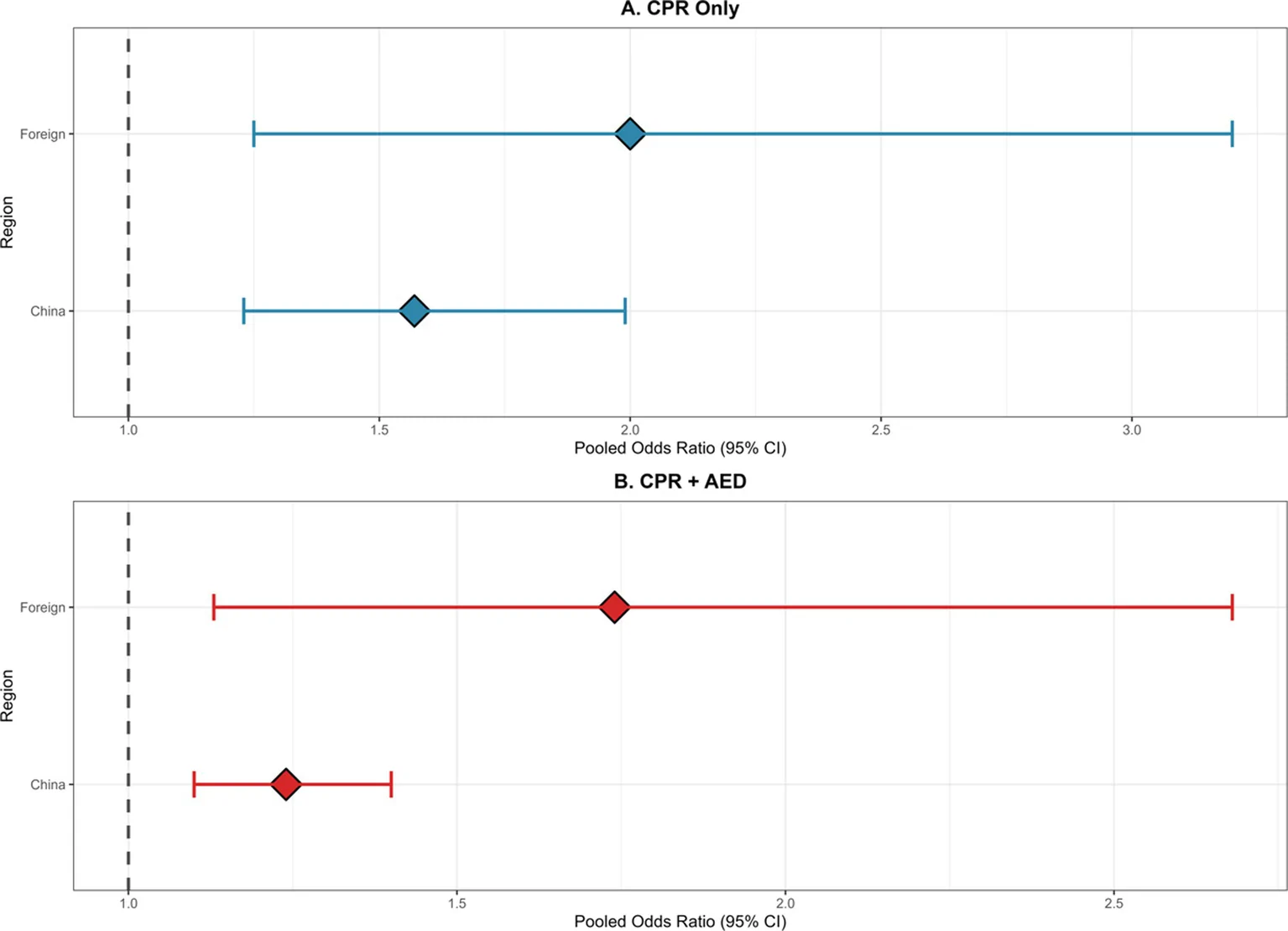
Subgroup analysis of the association between layperson CPR training and survival outcomes according to geographic region. **(A)** CPR-only training. **(B)** CPR plus AED training. Pooled estimates were calculated using random-effects models.

The results for the subgroup analyses, based on geographic region, are shown in Table 4 and Figure 4. Favorable associations with survival were observed in both Chinese and non-Chinese settings, although the magnitude of the pooled estimates differed between regions. For CPR-only training, non-Chinese studies showed a higher pooled effect (OR = 2.00, 95% CI: 1.25–3.20) compared with Chinese studies (OR = 1.57, 95% CI: 1.23–1.99). The same trend was seen for training on CPR with AED (non-China: OR = 1.74 vs. China: OR = 1.24). Substantial heterogeneity also persisted within several geographic subgroups, indicating that classification as Chinese or non-Chinese alone did not fully explain the variation in study-specific estimates. These patterns may reflect broader differences in OHCA systems, including baseline bystander CPR rates, EMS response intervals, population training coverage, AED accessibility, and the maturity of public health and resuscitation infrastructure.

**Table 4:** Subgroup analysis of the association between CPR training and survival outcomes by geographic region.

Training modality	Region	No. of studies	No. of participants	Q	I^2^ (%)	*P* for heterogeneity	Pooled OR (95% CI)	*Z*	*p*
CPR-only training	China	4	42,797	16.104	81.4	0.0011	1.57 (1.23–1.99)	3.68	< 0.001
CPR-only training	Non-China	6	56,941	35.713	86.0	<0.001	2.00 (1.25–3.20)	2.88	0.004
CPR plus AED training	China	2	4,693	0.067	13.93	0.795	1.24 (1.10–1.40)	3.45	<0.001
CPR plus AED training	Non-China	5	40,344	19.321	79.3	<0.001	1.74 (1.13–2.68)	2.52	0.012

OR, odds ratio; CI, confidence interval; CPR, cardiopulmonary resuscitation; AED, automated external defibrillator. Estimates were obtained using random-effects models.

#### 3.4.2 Temporal variation in the observed associations

**Figure 5:**
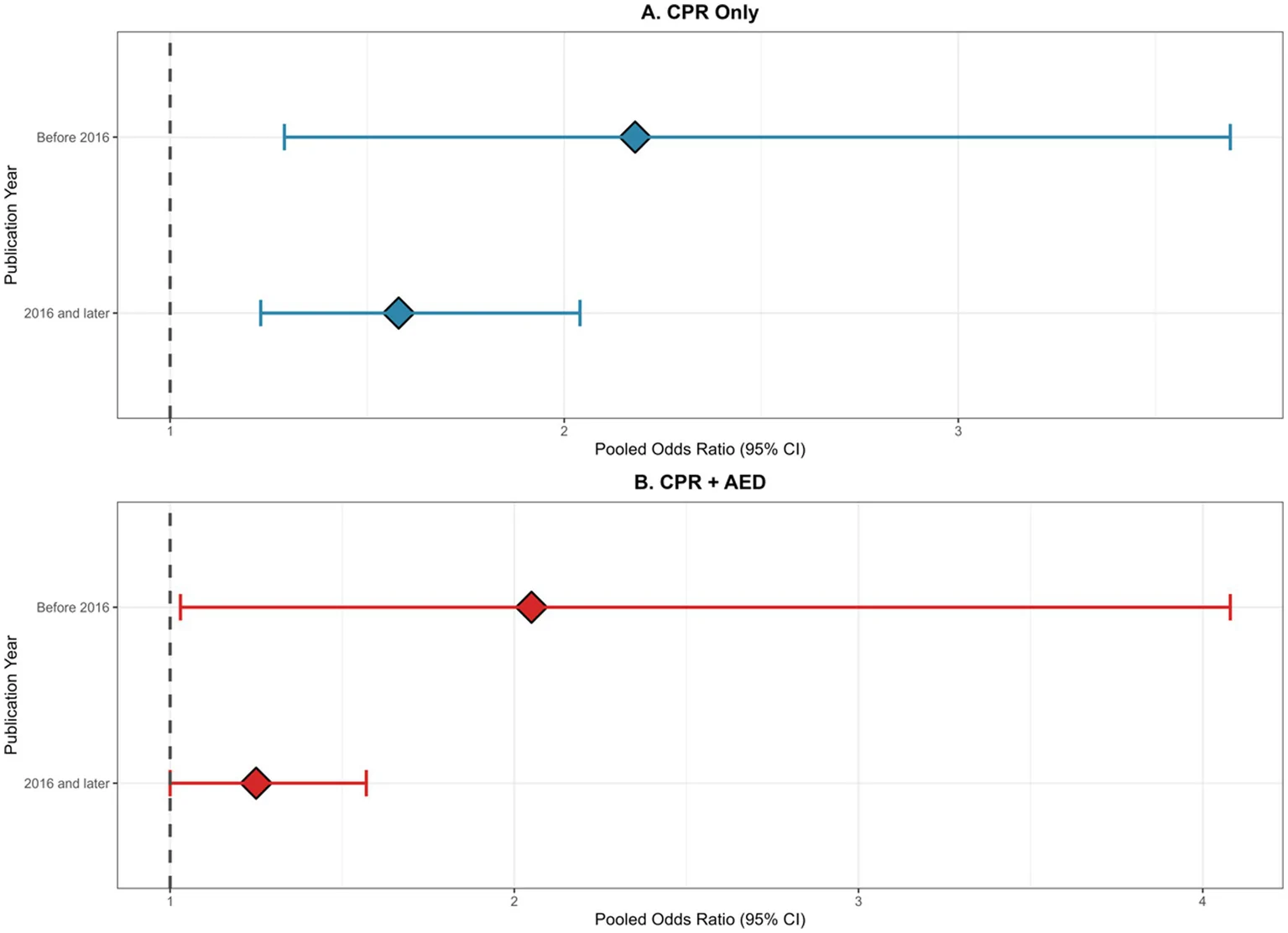
Subgroup analysis of the association between layperson CPR training and survival outcomes according to publication period. **(A)** CPR-only training. **(B)** CPR plus AED training. Studies were stratified by publication period (before 2016 vs 2016 and later), with pooled estimates calculated using randomeffects models.

Table 5 and Figure 5 show the results of the subgroup analysis by publication year. The magnitude of the pooled association was lower in more recently published studies for both training categories. For CPR-only training, earlier studies (≤2016) demonstrated stronger effects (OR = 2.18, 95% CI: 1.29–3.69), whereas more recent studies (>2016) showed lower but still significant effects (OR = 1.58, 95% CI: 1.23–2.04). A similar pattern was observed for CPR plus AED training: studies published in or before 2016 showed a larger association (OR = 2.05, 95% CI: 1.03–4.08), whereas studies published after 2016 showed a smaller association that did not reach conventional statistical significance (OR = 1.25, 95% CI: 1.00–1.57; *p* = 0.054). This temporal pattern may partly reflect improvements in baseline resuscitation systems, including greater public awareness of CPR, wider implementation of dispatcher-assisted CPR, and stronger emergency response infrastructure, which may reduce the additional association observed after the introduction of new training programmes.

**Table 5:** Subgroup analysis of the association between CPR training and survival outcomes by publication period.

Training modality	Publication period	No. of studies	No. of participants	Q	I^2^ (%)	*p* for heterogeneity	Pooled OR (95% CI)	*Z*	*p*
CPR-only training	≤2016	4	48,678	28.293	89.4	< 0.001	2.18 (1.29–3.69)	2.90	0.004
CPR-only training	>2016	6	51,060	23.825	79.0	< 0.001	1.58 (1.23–2.04)	3.54	< 0.001
CPR plus AED training	≤2016	3	3,246	11.276	82.3	0.004	2.05 (1.03–4.08)	2.05	0.040
CPR plus AED training	>2016	4	41,791	8.471	64.6	0.037	1.25 (1.00–1.57)	1.93	0.054

OR, odds ratio; CI, confidence interval; CPR, cardiopulmonary resuscitation; AED, automated external defibrillator. Estimates were obtained using random-effects models.

### 3.5 Sensitivity analysis and robustness of pooled estimates

**Figure 6:**
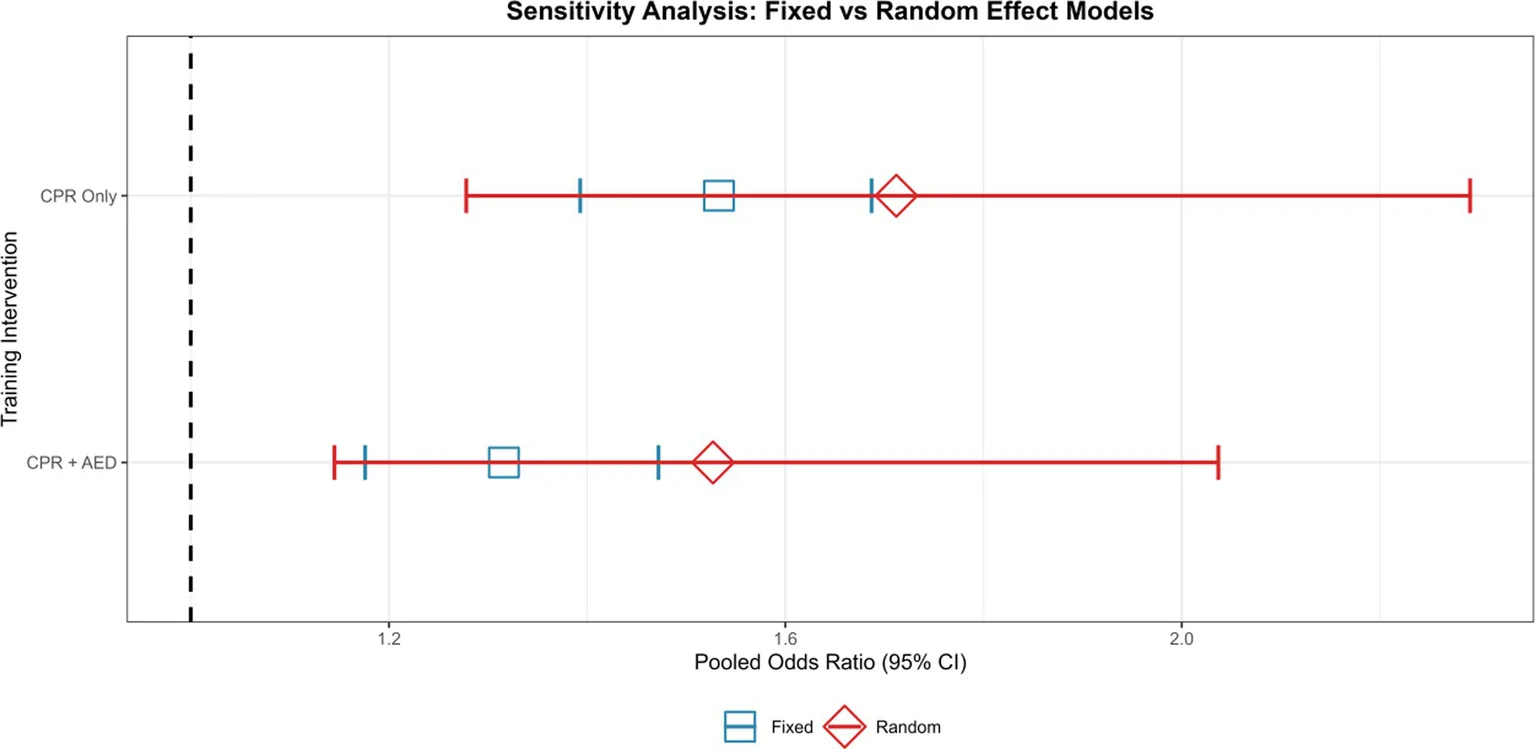
Leave-one-out sensitivity analysis of the pooled associations between CPR training and OHCA survival.

Both fixed-effect and random-effects models were used to perform sensitivity analyses to assess the robustness of the results (Table 6; Figure 6). The pooled effect estimates did not change in the various analytic approaches. CPR only training ORs were between 1.53 and 1.71, and CPR plus AED training was between 1.32 and 1.53. Importantly, none of the CIs were below unity and none of the individual studies had a disproportionate influence on the total effect size, thus affirming the robustness of the meta-analytic estimates.

**Table 6:** Sensitivity analysis comparing fixed-effect and random-effects models.

Training modality	Fixed-effect OR (95% CI)	*p* value	Random-effects OR (95% CI)	*p* value
CPR-only training	1.533 (1.393–1.687)	<0.001	1.712 (1.278–2.291)	<0.001
CPR plus AED training	1.316 (1.176–1.472)	<0.001	1.527 (1.145–2.037)	0.0039

OR, odds ratio; CI, confidence interval; CPR, cardiopulmonary resuscitation; AED, automated external defibrillator.

### 3.6 Publication bias and small-study effects

**Figure 7:**
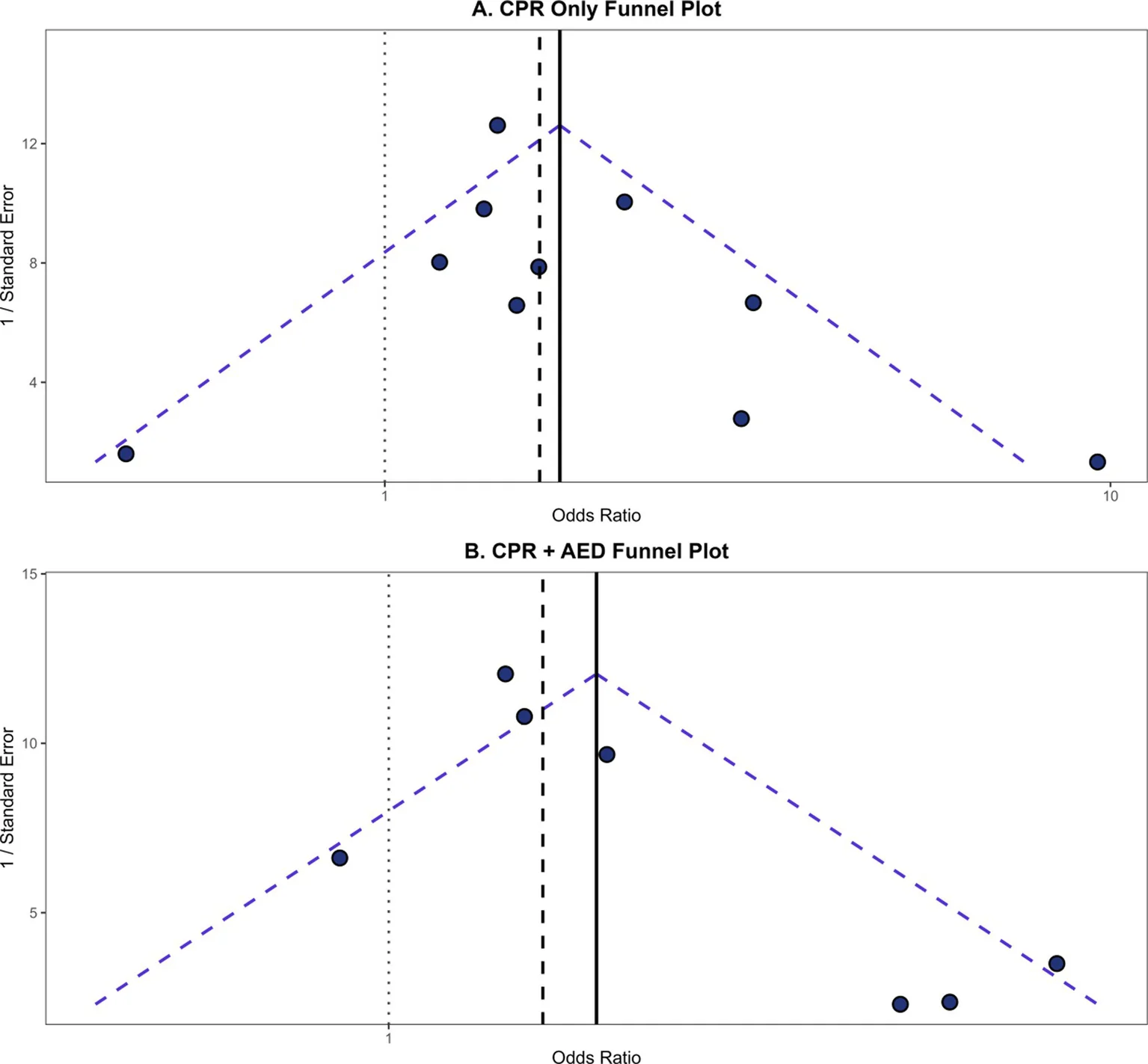
Publication bias assessment using funnel plot and Egger–Begg tests. **(A)** CPR only funnel plot. **(B)** CPR + AED funnel plot.

Egger’s regression test and Begg’s rank correlation test were used to evaluate small-study effects (Table 7; Figure 7). There was no statistically significant publication bias in either CPR only or CPR plus AED group (all *p* > 0.05). Funnel plots were inspected visually and there was no strong evidence of small-study effects or selective reporting bias. These analyses did not identify strong statistical evidence of small-study effects, although their power was limited by the number of studies in each analysis.

**Table 7:** Assessment of small-study effects using Egger’s and Begg’s tests.

Training modality	No. of studies	Egger value	Egger *p* value	Begg *Z*	Begg *p* value
CPR-only training	10	0.890	0.069	−0.699	0.484
CPR plus AED training	7	0.685	0.144	−1.178	0.239

CPR, cardiopulmonary resuscitation; AED, automated external defibrillator.

## 4 Discussion

This systematic review and meta-analysis identified a population-level association between layperson CPR training and higher survival after OHCA. Across 19 studies conducted in diverse healthcare and socioeconomic settings, training-related strategies were generally associated with more favorable survival outcomes. The consistency of the observed association across different settings highlights the potential relevance of layperson CPR training within community and regional resuscitation systems.

The observed association with survival is biologically consistent with the time-dependent cerebral and myocardial ischemia that occurs during cardiac arrest. In absence of circulation, irreversible neuronal damage starts after a few minutes and early intervention is crucial (27). CPR training may increase the likelihood of immediate bystander intervention, thereby shortening the no-flow interval and supporting minimal perfusion until advanced care or defibrillation becomes available (28). Significantly, this is not just a physiological process but also a behavioural one, and an intervention requires the bystander to be able to recognise arrest, overcome any hesitation and be able to start resuscitation under stress (29). CPR training, in this context, is a cognitive-behavioral trigger to move from passive to active response.

This mechanism is system level support based on evidence from large population-based studies. Wissenberg et al. (10) have shown that bystander CPR rates were associated with a significant and sustained improvement in survival after OHCA in nationwide initiatives in Denmark, indicating that it is crucial to have coordinated national resuscitation strategies instead of individual interventions. Likewise, in a large regional cohort, Malta Hansen et al. (11) found that uptick in bystander intervention was independent of other factors and associated with the likelihood of OHCA survival, further confirming the importance of early-bystander-response in OHCA outcomes. Taken together, the available evidence suggests that the association between CPR training and survival may be mediated partly by a greater likelihood of early bystander intervention and by improvements in the timeliness or quality of initial resuscitation.

At the system level, Bobrow et al. (30) reported that a community-wide resuscitation strategy incorporating dispatcher assistance, EMS optimization, and CPR training was associated with higher OHCA survival. Hasselqvist-Ax et al. (31) similarly found that early bystander CPR was associated with a higher likelihood of survival than no CPR or delayed CPR. These studies place the association observed in the present meta-analysis within a broader resuscitation pathway: training may facilitate recognition and initial bystander action, while subsequent outcomes also depend on dispatcher support, defibrillation, EMS performance, advanced life support, and post-resuscitation care.

CPR training primarily targets the earliest links in the chain of survival by preparing laypersons to recognize cardiac arrest, activate EMS, and initiate chest compressions. Survival to hospital discharge, however, reflects the cumulative performance of the entire resuscitation system. Timely AED use, dispatcher-assisted CPR, rapid EMS arrival, high-quality advanced life support, appropriate transport decisions, coronary and critical care interventions, temperature management, and neurological prognostication may all influence the final outcome. The population-level association attributed to CPR training should therefore be interpreted within this linked sequence of care.

The substantial heterogeneity observed across studies likely reflects meaningful differences in the populations, training strategies, study methods, and resuscitation systems evaluated (32). Training programmes varied in content, intensity, duration, delivery method, target population, refresher frequency, population coverage, and integration with AED education or dispatcher support. The participating regions also differed in baseline bystander CPR rates, EMS response intervals, dispatcher-assisted CPR protocols, AED accessibility, public awareness, regional resuscitation policies, and the organization of advanced life support and post-resuscitation care. Methodological differences in study design, exposure ascertainment, outcome definitions, follow-up periods, and covariate adjustment may have contributed further to variation in the study-specific estimates. The persistence of heterogeneity within several geographic and temporal subgroups indicates that no single study-level classification fully accounted for the observed variation. Collectively, these findings indicate that the association between CPR training and survival is closely related to the wider resuscitation environment (e.g., registry-based studies of OHCA survival showed that cumulative improvements across multiple links in the chain of survival were responsible for the improvements in survival, but not any individual intervention).

CPR training can be thought of as an intervention that reduces the “activation threshold” for emergency response, in behavioral terms. It enhances recognition and decreases the perceived legal and psychological obstacles, as well as raising self-efficacy during high stress situations (33). Blewer et al. (20) reported that community-based CPR education was associated with higher bystander CPR rates and more favorable survival outcomes, supporting a measurable population-level change in emergency-response behaviour. These findings are consistent with CPR training functioning both as skill acquisition and as preparation for rapid behavioural activation during emergencies.

The difference between the pooled estimates for CPR-only training and CPR plus AED training should be interpreted in relation to system readiness rather than as evidence of superiority of one strategy. Both approaches were associated with higher survival, while variation in AED density, accessibility, retrieval time, and actual use may have influenced the magnitude of the observed associations. AED use is particularly relevant for shockable rhythms, but its population-level contribution depends on device density, accessibility, retrieval time, and actual deployment. CPR training addresses the more broadly applicable initial bystander response, whereas AED education represents an additional component whose contribution varies with the readiness of the local public-access defibrillation system.

Moreover, the results of our meta-analysis support the overall literature that CPR by dispatchers has been shown to be a beneficial intervention for survival. Dispatcher-supported interventions have been associated with greater delivery of bystander CPR and more favorable survival outcomes (34). Real-time dispatcher guidance may complement prior training by reducing hesitation and providing structured instructions during an emergency.

The design and complexity of CPR instruction may also influence the uptake and application of layperson training. In a recent systematic review and meta-analysis, Hasanvand et al. (35) reported broadly comparable overall survival and neurological outcomes between compression-only CPR and conventional CPR in adults with OHCA. These findings support compression-only CPR as a simplified and practical option for layperson education, particularly in settings where reducing procedural complexity may facilitate wider participation and earlier bystander intervention.

The influence of subsequent pre-hospital management is further illustrated by Shimomura et al. (36), who compared earlier intra-arrest transport with continued on-scene resuscitation in a nationwide OHCA cohort. The association with survival differed according to the first documented rhythm: survival was similar between strategies among patients with shockable rhythms, whereas continued on-scene resuscitation was associated with more favorable survival among patients with non-shockable rhythms. These findings highlight that transport timing, initial rhythm, and rhythm-specific pre-hospital strategy may influence outcomes after initial bystander CPR has been initiated.

A temporal decline in effect size in more recent studies could be due to better baseline survival rates with improved EMS systems, CPR awareness and widespread dissemination of resuscitation guidelines. It could also reflect methodological improvements in more recent studies, frequently employing larger data sets and better adjustment for confounding factors and providing more conservative estimates. In this sense, these trends may indicate that CPR training is increasingly being introduced in a survival system that already is enhanced, and the “return on investment” of CPR will be spread more widely across a more efficient system.

In a public health context, CPR training should be viewed as a foundational component of population emergency preparedness rather than as a stand-alone educational programme. Its contribution is integrated with rapid emergency-call recognition, dispatcher-assisted CPR, timely EMS response, accessible AEDs, advanced life support, and organized post-resuscitation care. Mandatory school-based CPR programmes may support early acquisition of life-saving skills and sustained population coverage. Equitable access to training and resuscitation resources is also important because socioeconomic differences in training opportunities, EMS access, and AED availability may contribute to disparities in OHCA outcomes.

Several limitations should be acknowledged. First, the included studies were observational or non-randomized, and residual confounding related to EMS performance, baseline bystander CPR rates, socioeconomic conditions, AED availability, initial cardiac rhythm, arrest location, regional resuscitation policies, concurrent public health initiatives, and post-resuscitation care cannot be excluded. In several studies, CPR training was introduced alongside broader changes in dispatcher protocols, AED deployment, EMS organization, or public health policy, and the independent contribution of training could not always be isolated from these concurrent system-level changes. The extent and selection of covariate adjustment also varied across studies, and estimates calculated from unadjusted event data may have been more susceptible to confounding than multivariable-adjusted estimates. The risk-of-bias assessment should also be interpreted with appropriate caution. Although the NOS enabled a consistent evaluation of the predominantly cohort- and registry-based evidence, its summary scoring approach may obscure domain-specific concerns, and some items involve subjective judgment. Second, differences in training exposure, outcome definitions, and healthcare systems contributed to substantial between-study heterogeneity; the pooled estimates should therefore be interpreted as average associations across different resuscitation settings. The available number of studies and consistently reported study-level characteristics did not support a reliable meta-regression analysis of individual moderators. Third, although survival to hospital discharge was prioritized, some studies reported 30-day survival or another closely related longer-term survival measure; outcome definitions and follow-up time points were therefore not fully uniform across studies. Finally, although the statistical tests did not indicate significant publication bias, their power was limited by the number of studies, and selective reporting cannot be completely excluded.

Overall, population-level layperson CPR training was associated with higher survival after OHCA and should be viewed as an important, modifiable component of an integrated resuscitation system rather than as a stand-alone strategy. Its contribution occurs within a coordinated pathway that includes early cardiac arrest recognition, dispatcher assistance, rapid EMS response, timely access to AEDs, advanced life support, and high-quality post-resuscitation care. Meta-regression analyses to quantify system-level effect modifiers and to examine the interaction between training intensity and emergency infrastructure for survival outcomes should be the focus of future research.

## Data Availability

Publicly available datasets were analyzed in this study. This data can be found here: All data generated or analysed during this study are included in the published articles, and their references are listed in the reference section.
